# Enhancing Mask Transformer with Auxiliary Convolution Layers for Semantic Segmentation

**DOI:** 10.3390/s23020581

**Published:** 2023-01-04

**Authors:** Zhengyu Xia, Joohee Kim

**Affiliations:** Department of Electrical and Computer Engineering, Illinois Institute of Technology, Chicago, IL 60616, USA

**Keywords:** deep learning, semantic segmentation, image segmentation, transformer, convolutional neural networks

## Abstract

Transformer-based semantic segmentation methods have achieved excellent performance in recent years. Mask2Former is one of the well-known transformer-based methods which unifies common image segmentation into a universal model. However, it performs relatively poorly in obtaining local features and segmenting small objects due to relying heavily on transformers. To this end, we propose a simple yet effective architecture that introduces auxiliary branches to Mask2Former during training to capture dense local features on the encoder side. The obtained features help improve the performance of learning local information and segmenting small objects. Since the proposed auxiliary convolution layers are required only for training and can be removed during inference, the performance gain can be obtained without additional computation at inference. Experimental results show that our model can achieve state-of-the-art performance (57.6% mIoU) on the ADE20K and (84.8% mIoU) on the Cityscapes datasets.

## 1. Introduction

Transformer, a type of a deep learning model based on self-attention [1], was first applied to natural language processing (NLP) tasks and achieved significant improvements. Inspired by the huge success of Transformer architectures in NLP, extensive research has been recently performed to apply Transformer to various computer vision tasks [2,3,4]. The basic idea for vision transformers is to break down images into sequential patches and learn self-attention features without using convolutional layers. Unlike traditional convolutional neural network (CNN) models [5,6], transformer-based ones can better capture global attention and broader range relations throughout the entire layers.

Recently, several semantic segmentation approaches [7,8,9,10,11] based on vision transformers have been proposed to exploit the benefits of transformer models for improving semantic segmentation. One way to improve semantic segmentation is to adopt a feature pyramid network (FPN) [12] in a transformer model to obtain multi-scale feature maps. For example, SETR [7] designs a top-down feature aggregation at the decoder side. It generates the final predictions by collecting the feature maps from the transformer backbone. SegFormer [8] proposes a hierarchical transformer at the encoder side. The feature outputs are then fused into a multilayer perceptron (MLP) decoder to aggregate information. Another way is to replace per-pixel classification with mask classification to predict the final outputs. Segmenter [9] utilizes a transformer-based decoder to generate class masks by computing the scalar product between the patch embeddings and the class embeddings. MaskFormer [10] observes that mask classification is sufficiently general to solve both semantic- and instance-level segmentation tasks. It converts per-pixel classification into a mask classification model using a set prediction mechanism. Mask2Former [11] improves the performance on top of [10] and presents a universal segmentation model using the same mask classification mechanism.

However, we observe that these segmentation approaches rely heavily on transformer models and therefore lose local information at a certain level. Even Mask2Former [11], a powerful unified segmentation model, still faces the issue of learning local features and segmenting small objects. In contrast, convolutional layers can better capture local features since most CNN models adopt a small window-sized learning manner. In addition, the optimization with CNN models is easier and more robust compared to transformer models. Therefore, many researchers consider a hybrid model which combines the benefits of CNNs and transformers. For example, ref. [13] replaces the ViT patching module with a convolutional stem to achieve faster convergence and more stable training. BotNet [14] incorporates multi-head self-attention modules on top of the ResNet. It provides a backbone architecture that uses transformer-like blocks for downstream tasks. Visformer [15] offers an empirical study by transforming a transformer-based model to a CNN model and then proposes a new hybrid architecture by absorbing the advantages and discarding the disadvantages.

Inspired by these hybrid approaches, we propose a simple yet effective method on top of [11] to boost semantic segmentation performance. In this work, we introduce an auxiliary CNN on the encoder side. It encourages the model to learn dense local features compared to a pure transformer-based backbone. Additionally, unlike the existing hybrid models, our proposed auxiliary convolution layers can be removed. Therefore, it enhances the semantic segmentation performance without any additional computational cost at inference. Since [11] is a universal segmentation model, we will also show that our proposed method can improve the semantic segmentation performance using a single panoptic model. The contributions of this work can be summarized as follows:

(1) We design an auxiliary CNN on top of Mask2Former [11] to help improve semantic segmentation performance. The proposed network consists of simple convolutional layers without bells and whistles. We demonstrate that the proposed method improves the semantic segmentation performance quantitatively and qualitatively. Specifically, we show that the proposed method is effective in learning local features and segmenting small objects more accurately.
(2) Since the proposed auxiliary convolution layers are required during the training stage only, the proposed method incurs no additional computation overhead at inference. This is one of the important properties of the proposed method because enhancing the performance while maintaining the complexity at inference is crucial for real-world applications.
(3) The proposed auxiliary convolution layers are effective for both semantic and panoptic segmentation. Since Mask2Former is a universal architecture for different segmentation tasks and our proposed method is designed to enhance Mask2Former, we show that the proposed method achieves state-of-the-art performance for semantic and panoptic segmentation on the ADE20K [16] and Cityscapes [17] datasets.

The rest of the paper is organized as follows: In Section 2, the related work is discussed. In Section 3, the proposed method is explained in detail. Section 4 introduces the dataset, implement details, ablation study and experimental results. Section 5 is the conclusion and future work.

## 2. Related Work

### 2.1. Semantic Segmentation

Semantic segmentation aims to assign a category label to each pixel. Ref. [18] is the first work to train a fully convolutional network (FCN) end-to-end for semantic segmentation. SegNet [19] and UNet [20] extend the segmentation model with a symmetric encoder-decoder architecture to gradually recover image resolutions. ParseNet [21] augments the features with the average feature for each layer to exploit global context information. PSPNet [22] and DeepLab [23,24,25] follow the ideas of Spatial Pyramid Pooling (SPP) [26] to capture dense contextual information at multiple levels. DANet [27] appends two separate attention modules on top of FCN to obtain global dependencies in spatial and channel dimensions, respectively. CCNet [28] proposes a criss-cross attention module on the decoder side to harvest contextual information along the criss-cross path. OCNet [29] presents an object context aggregation scheme with an interlaced spare self-attention to address the semantic segmentation task. These well-known models are all based on convolutional neural networks to learn image features. With the advent of vision transformers [2,3,4], many semantic segmentation approaches are proposed based on transformers. SETR [7] reformulates semantic segmentation as a sequence-to-sequence learning problem and deploys a pure encoder-decoder transformer model for semantic segmentation. SegFormer [8] designs a hierarchical transformer encoder with a lightweight MLP decoder to generate segmentation results without heavy computational cost. Segmenter [9] refers to ViT [2] and extends it to semantic segmentation. It adopts a mask transformer on the decoder side to generate class masks.

### 2.2. Panoptic Segmentation

Panoptic segmentation [30] aims to combine semantic and instance segmentation into a general unified output. Panoptic-Deeplab [31] and TASCNet [32] build one shared backbone with two segmentation heads to learn semantic and instance features individually. UPSNet [33] designs a parameter-free panoptic head using pixel-wise classification to resolve the conflicts between semantic and instance features. BGRNet [34] adopts a graph structure on top of a panoptic network to mine intra- and inter-modular relations between foreground and background classes. Auto-Panoptic [35] proposes an automated framework to search for main components simultaneously in a panoptic network, achieving a reciprocal relation between things and stuff classes. Panoptic-FCN [36] represents things and stuff uniformly using a proposed kernel head, which generates unique weights for both classes. MaskFormer [10] demonstrates that mask classification is sufficient to be used for both semantic- and instance-level segmentation tasks. It shows that a simple mask classification can outperform state-of-the-art per-pixel classification models. Mask2Former [11] is an improved version of [10] and utilizes masked attention to extract localized features. It is a universal image segmentation model that outperforms specialized segmentation models across different tasks.

### 2.3. Hybrid Models Using Convolutions and Transformers

Recently, numerous approaches that combine both convolutions and transformers have been proposed. DETR [26] adopts a CNN backbone with a transformer decoder for object detection. ViLBERT [37] builds a multimodal two-steam model to process visual and textual inputs through co-attentional transformer layers. It utilizes a BERT [4] architecture for the linguistic stream and a Faster-RCNN [38] to capture image regions. PVT [39,40] borrows the pyramid structure concept in CNNs and designs a pyramid vision transformer for learning multi-scale features with high resolutions. P2T [41] implements a pooling-based self-attention module with depthwise convolutional operations for multi-scale feature learning. Ref. [13] demonstrates that the optimization challenges in ViT [2] are related to the patchify stem and shows that the use of convolutional stem enables a much faster convergence in training. BotNet [14] and Visformer [15] analyze the behaviors in convolution- and transformer-based models. Both methods incorporate Multi-Head Self-Attention (MHSA) modules on top of the ResNet-like models to improve the performance of the baseline models. In this work, we propose a simple yet efficient method that introduces an auxiliary CNN on top of the Mask2Fomer [11]. It helps increase the semantic segmentation performance, especially for the local features and small objects. Unlike the existing hybrid models, the proposed method can be removed at the inference stage and therefore does not incur any additional computation overhead at inference.

## 3. Proposed Method

### 3.1. Overall Architecture

**Figure 1:**
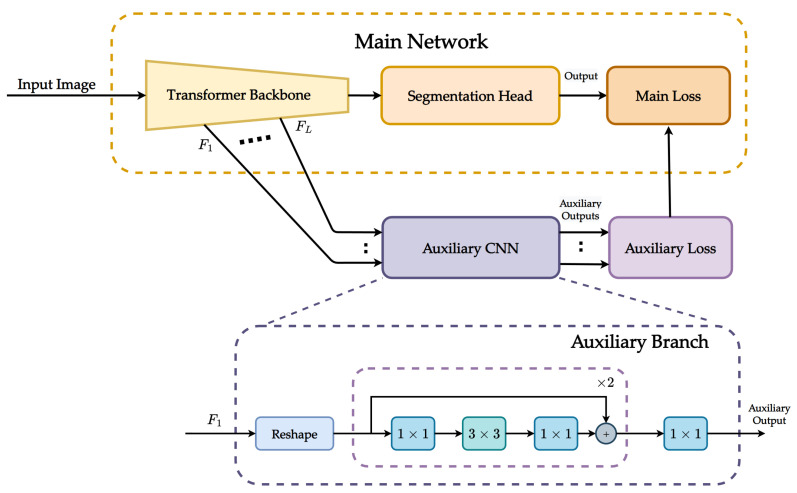
Architecture overview. The proposed method is instantiated on top of Mask2Former [11], which uses Swin Transformer [42] as the backbone network to extract feature embeddings $\left\{F_{1},\ldots ,F_{L}\right\}$. The proposed auxiliary CNN consists of several simple convolutional layers to learn more accurate local features by using the feature embedding produced by the Transformer backbone as input. An auxiliary loss is used along with the main loss to compute the total loss for segmentation. The auxiliary CNN is used for training only and will be removed at inference.

Our proposed method is integrated with the transformer-based model to improve semantic segmentation. Figure 1 illustrates the overall architecture, where the proposed auxiliary CNN is jointly trained with the main segmentation network. First, the input image is fed to a Swin [42] backbone to generate feature embeddings $F_{l}$, where l∈{1,…,L} and *L* is the total number of the stages represented in the Swin backbone. Then, feature embeddings $F_{l}$ are shared between two separate branches: the main segmentation head and the proposed auxiliary CNN. We use Mask2Former [11] which adopts a pixel decoder and a transformer decoder to generate mask predictions as the main segmentation head. In the auxiliary CNN, feature embeddings $F_{l}$ are first fed to a simple CNN-based network, aiming to learn local features with different resolutions. Then, an auxiliary loss is calculated based on the auxiliary outputs and added to the main loss to compute the total loss.

### 3.2. Auxiliary CNN

We design an auxiliary branch with convolutional layers to generate multi-scale local features from the backbone, as illustrated in Figure 1.

For a feature embedding $F_{l}\in R^{\frac{HW}{r_{l}^{2}}\times C_{l}}$, we first reshape it into a feature map $F_{l}^{{}^{\prime }}$ with a size of $C_{l}\times \frac{H}{r_{l}}\times \frac{W}{r_{l}}$, where $C_{l}$ is the channel dimension of the feature map at the *l*th stage in the Swin backbone. *H* and *W* are the height and width of the input image, respectively. $r_{l}$ is the resolution factor equal to 4, 8, 16, and 32 for stages 1, 2, 3, and 4, respectively. Then, the reshaped feature map $F_{l}^{{}^{\prime }}$ is applied to a series of residual blocks for local feature learning. The residual block consists of a stack of three convolutional layers. The three layers are 1×1, 3×3, and 1×1 convolutions, where the 1×1 layers are responsible for downsampling and upsampling the channel dimensions and 3×3 filters are used for feature learning. A skip connection and an element-wise summation are included in the residual block to refine the optimization processing during the training phase. Then, the output from the residual block is fed to a 1×1 convolutional layer to reduce the feature dimension from $C_{l}\times \frac{H}{r_{l}}\times \frac{W}{r_{l}}$ to $N\times \frac{H}{r_{l}}\times \frac{W}{r_{l}}$, where *N* is the number of categories in the dataset. Finally, we adopt a cross-entropy function for auxiliary loss calculation. Note that the reshaping operation in the proposed method is not mandatory. Depending on the shape of the output obtained from the Transformer backbone, the proposed auxiliary CNN can be used without reshaping the feature embeddings.

### 3.3. Auxiliary Loss

We define the loss function for auxiliary CNN as a cross-entropy loss. Specifically, the loss function for auxiliary branch at the *l*th stage is computed as:

(1) $$L_{aux}^{l}=\sum_{w=1}^{W^{l}}\sum_{h=1}^{H^{l}}CE\left(y^{l}\left(w,h\right),gt^{l}\left(w,h\right)\right),$$

where $W^{l}=W/r_{l}$ and $H^{l}=W/r_{l}$. $y^{l}$ is the auxiliary prediction at the *l* stage, and $gt^{l}$ is the corresponding ground truth for semantic segmentation. CE is the cross-entropy loss function. The total auxiliary segmentation loss $L_{aux}^{}$ is the normalized sum of the cross-entropy loss $L_{aux}^{l}$ over all *L* stages and is defined as:

(2) $$L_{aux}=\sum_{n=1}^{L}norm\left(L_{aux}^{l}\right).$$

When training with the auxiliary CNN, the total loss function is defined as:

(3) $$L_{total}=L_{mask-cls}+\beta L_{aux},$$

where $L_{mask-cls}$ is the mask classification loss defined in [11], β is the weight for auxiliary segmentation loss. In our ablation study, the best β is selected as 0.1.

## 4. Experimental Results

### 4.1. Dataset

We conduct experiments on the ADE20K [16] and Cityscapes [17] datasets. The ADE20K dataset is a densely annotated dataset for scene parsing with 150 categories. The training set contains 20K images, and the validation set contains 2K images. The Cityscapes dataset is a street-view dataset with 19 classes, focusing on a semantic understanding of urban street scenes. It contains 5K images with fine annotations and 20K images with coarse annotations. The fine-annotated dataset contains 2975, 500, and 1525 images for training, validation, and testing, respectively. The ADE20K validation dataset is used for the ablation study to compare the performance with our baseline Mask2Former [11] and other setups.

We use the mean Intersection-over-Union (mIoU) metric for semantic segmentation and the standard Panoptic Quality (PQ) metric for panoptic segmentation. PQ metric [30] evaluates the performance of both stuff and things in a unified manner. Additionally, we use the same metric settings for semantic and instance segmentation based on a single panoptic model as in [11]. Specifically, we report mIoU_pan_ for semantic segmentation by merging instance masks with the same category, and AP_pan_ for instance segmentation, evaluated on the “thing” categories with instance segmentation annotations.

### 4.2. Implementation Details

Our implementation is based on PyTorch [43] framework with Detectron2 [44]. We use the AdamW [45] optimizer and the step learning rate schedule, where the base learning rate is initialized to 0.0001. All the training has a weight decay of 0.05 and a momentum of 0.9. The input image is resized to 640×640 and 512×1024 for ADE20K and Cityscapes, respectively. Data augmentation includes random crop, random flip, and large-scale jittering (LSJ) [46]. Following the default settings in [11], we adopt batch normalization for the Cityscapes dataset only. The query number is 100 for all training except that we set 200 queries for the panoptic model with the Swin-L backbone.

Due to the GPU memory limitation, we use smaller batch sizes with higher numbers of training iterations so that we can have similar training settings as in Mask2Former. Specifically, for the ADE20K dataset, we set the batch sizes to 16, 16, 12, and 8 for the Swin-T, Swin-S, Swin-B, and Swin-L transformer backbones, respectively. The corresponding training iterations for these Swin transformer backbones are set to 160K, 160K, 240K, and 360K, respectively. For the Cityscapes dataset, we assign the batch sizes to 12, 8, and 6 for the Swin-S, Swin-B, and Swin-L, respectively. The corresponding training iterations are set to 120K, 180K, and 240K, respectively. By doing so, the number of training epochs is the same as [11]. We also represent the reproduced Mask2Former with our settings, marked as Mask2Former(ours), for a fair comparison.

### 4.3. Ablation Study

We conduct our ablation study on the ADE20K validation dataset. To evaluate our proposed method fairly, we use the same experimental environments to compare the performance with different settings. We use the Mask2Former with Swin-B backbone as the base network. The cropping size of the input data is set to 640×640.

**Effectiveness of auxiliary CNN:** To determine the best architecture of the proposed auxiliary CNN for local feature learning, we first use different combinations of the multi-scale feature maps obtained from the Swin transformer backbone as input and evaluate the performance. Specifically, we set various setups by using $\left\{F_{1}\right\}$, $\left\{F_{2}\right\}$, $\left\{F_{3}\right\}$, $\left\{F_{1},F_{2}\right\}$, $\left\{F_{1},F_{3}\right\}$, $\left\{F_{2},F_{3}\right\}$, and $\left\{F_{1},F_{2},F_{3}\right\}$ as the feature inputs for our auxiliary branches. The subscript in $F_{l}$ indicates the stage number in the Swin backbone. Table 1 shows that the use of auxiliary CNN with any feature map generated from the transformer backbone improves the performance compared to the baseline method. Among all settings, the best performance is obtained when $\left\{F_{1},F_{2},F_{3}\right\}$ is used as input to the proposed auxiliary CNN. The experimental results verify that the proposed auxiliary CNN is effective in learning additional local features and achieves better performance when multi-scale features are used. Since the proposed auxiliary CNN will be removed at inference, we use $\left\{F_{1},F_{2},F_{3}\right\}$ as input to the proposed auxiliary CNN for the remaining experiments to achieve the best performance.

**Architecture of auxiliary CNN:** One of the main design criteria for the proposed auxiliary CNN is to learn some useful local information based on the feature maps generated from the transformer backbone network using simple architectures. We consider four different simple CNN architectures: a 1×1 convolutional layer, a 3×3 convolutional layer, a residual block (a stack of 1×1, 3×3, and 1×1 convolutional layers with a skip connection), and a stack of two residual blocks. Table 2 shows the comparison of performance gain in semantic segmentation obtained by using these different auxiliary CNN architectures for the ADE20K validation dataset. Among the simple architectures we considered, a stack of two residual blocks achieved the best performance improvement. Since stacking more than two residual blocks does not improve the performance gain significantly, we build our proposed auxiliary CNN by using a stack of two residual blocks.

**Weighting parameter of auxiliary CNN:** A weighting parameter β is introduced in Equation (3) to balance the loss between the main and the auxiliary tasks. The auxiliary CNN is trained along with the main segmentation network to enhance local features and improve segmenting small objects. However, while achieving this objective, the auxiliary task should not dominate the overall segmentation task. Table 3 shows the performance comparison when four different weighting parameters are used to adjust the contribution of the auxiliary loss. To maximize the overall performance by balancing the main and the auxiliary tasks, we set the weighting parameter β to 0.1.

### 4.4. Experimental Results for Semantic Segmentation

We compare the semantic segmentation performance of the proposed method with the recent transformer-based semantic segmentation models on the ADE20K and Cityscapes validation datasets. Since the performance of each model can be different from the one presented in the original paper depending on the hardware environment, we also include the performance of the baseline model Mask2Former obtained by our reproduced experiments.

For the ADE20K dataset, we can observe in Table 4 that our proposed method improves the performance of Mask2Former for all Swin Transformer backbones. Specifically, the proposed auxiliary CNN with Swin-T transformer backbone improves the baseline Mask2former by 0.9% and achieves 48.8% in mIoU (ss). With Swin-S, Swin-B^†^, and Swin-L^†^ Transformer backbones, the proposed method improves the mIoU by 0.9%, 0.4%, and 0.4%, respectively.

For the Cityscapes dataset, it can be seen from Table 5 that the proposed auxiliary CNN can enhance the Mask2Former’s semantic segmentation performance by 0.5%, 0.6% and 0.3% when Swin-S, Swin-B^†^, and Swin-L^†^ transformer backbones are used, respectively. Both experimental results show that our proposed method consistently outperforms Mask2Former with different Swin Transformer-based backbones. We observe that the performance with Swin-B is slightly better than with Swin-L. Two possible explanations for these results are: the use of smaller batch size for Swin-L in our experimental settings and the better multi-scale inference performance of Swin-B compared to Swin-L from the baseline.

**Figure 2:**
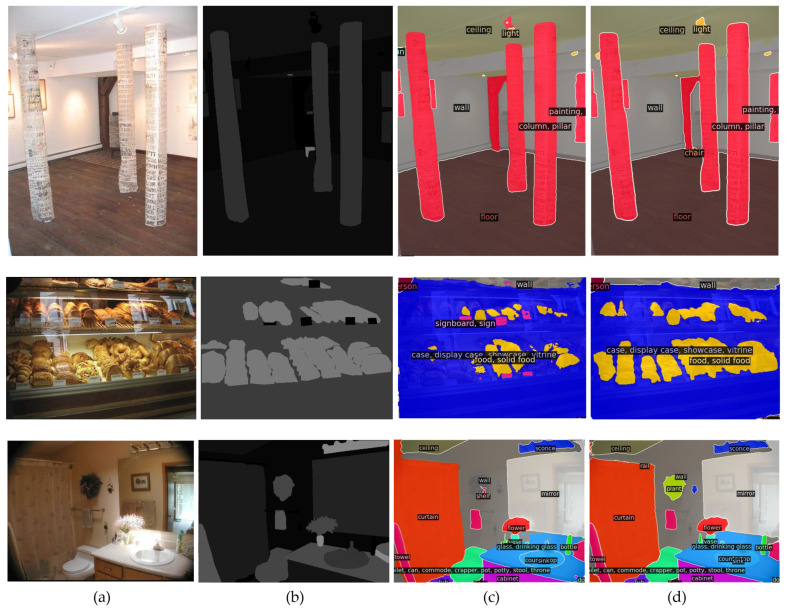
Semantic segmentation results based on the ADE20K validation dataset. (**a**) RGB input, (**b**) ground truth, (**c**) baseline method, and (**d**) our proposed method. The proposed method using the auxiliary CNN improves the detection of local information and small objects compared with the baseline method Mask2Former.

Since one of the main objectives of the proposed auxiliary CNN is to improve the segmentation performance in complex scenes which include small objects and require detailed local information for accurate segmentation, we show several qualitative results for the ADE20K and Cityscapes datasets. Figure 2 presents the qualitative results of the ADE20K validation dataset. In the first row, the category “light” on the ceiling is misclassified as a pillar by the baseline. Our proposed method can label the small object with the correct category. In the second row, the category “bread” labeled with khaki color is not segmented correctly using the baseline approach. However, our method can accurately segment most of them. In the third row, the baseline model fails to segment the category “plant” in the middle, while ours can detect and fully segment it.

**Figure 3:**
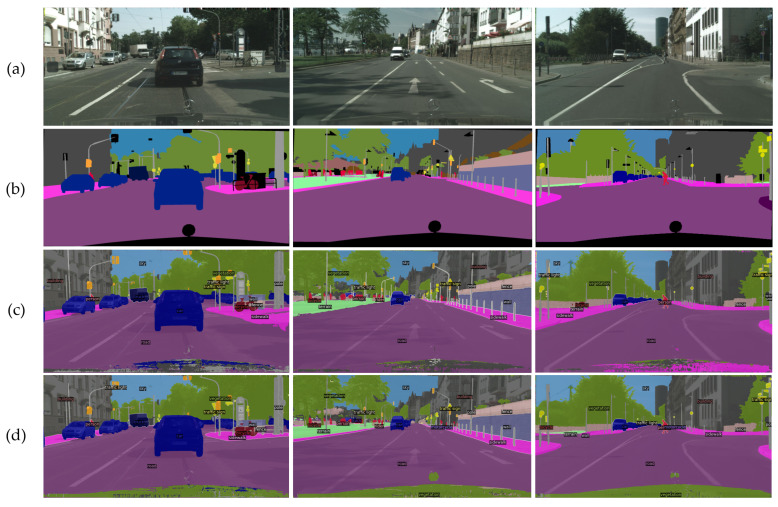
Semantic segmentation results based on the Cityscapes validation dataset. (**a**) RGB input, (**b**) ground truth, (**c**) baseline method, and (**d**) our proposed method. The proposed method using the auxiliary CNN improves the detection of local information and small objects compared with the baseline method Mask2Former.

Figure 3 shows the qualitative results of the Cityscapes validation dataset. In the first column, we can observe that the results generated by the baseline mislabeled the category “road” (labeled with purple) on the right-middle side as the category “sidewalk” (labeled with pink). Our proposed method can well distinguish both categories and segment them accurately. In the second column, the baseline approach cannot tell the difference between the category “terrain” (labeled with cyan) and “sidewalk” (labeled with pink), shown on the left side. As a result, the baseline erroneously merges both categories into one, while ours can correctly detect and segment these two categories. In the third column, we can observe that the baseline has difficulty detecting objects with similar textures on the left side. It recognizes the category “terrain” (labeled with cyan) and “road” (labeled with purple) as “sidewalk” (labeled with pink). Our proposed method can distinguish them clearly and accurately. The qualitative results prove that our proposed method can effectively learn local features and identify small objects much better than its baseline method.

### 4.5. Experimental Results for Panoptic Segmentation

Since our baseline method Mask2Former is a well-known universal segmentation model, we evaluate our proposed method using a single panoptic model. Again, since the hardware’s difference, we marked “Mask2Former(Ours)” as our reproduced results for the baseline method. Following the baseline’s settings, we set 100 queries for Swin-B backbone and 200 queries for Swin-L backbone.

The experimental results in Table 6 and Table 7 show that our proposed method can improve all segmentation performance. Specifically, we enhance the ADE20K’s panoptic, instance, and semantic segmentation performance with Swin-L backbone by 0.5%, 1.1%, and 0.3%, respectively. We also improve the panoptic, instance, and semantic segmentation performance for the Cityscapes dataset by 0.3%, 1.6%, and 0.3%, respectively. It proves that our proposed method can also improve all segmentation performance, even using a single panoptic model.

### 4.6. Limitations

The proposed method aims to adopt a simple auxiliary CNN on top of a transformer backbone to increase the overall segmentation performance. In Table 4 and Table 5, we can observe that the performance gain gradually decreases when the scale size of a Swin transformer backbone increases. It indicates that a fixed-size auxiliary CNN has less impact on a larger transformer. In our future work, we hope to design an auxiliary CNN that can be adaptive to the transformer backbones with different scales.

## 5. Conclusions

In this paper, we propose a simple yet effective auxiliary CNN architecture that introduces auxiliary convolutional layers to Mask2Former during training to learn dense local features. Since the proposed auxiliary CNN is required only for training and can be removed at inference, the segmentation performance can be improved without additional computation overhead at inference. Experimental results show that our proposed method achieves an mIoU of 57.6% on the ADE20K validation dataset and an mIoU of 84.8% on the Cityscapes validation dataset. In the future, we hope to develop a model that can be adaptive to the transformer backbones with different scales to improve the segmentation performance.

## Figures and Tables

**Table 1:** Performance comparison of different auxiliary CNN setups using the ADE20K validation set. baseline: Mask2Former with Swin-B backbone for semantic segmentation. $F_{l}$: feature embeddings extracted at the *l*th stage from the Swin-B backbone. ss: single-scale. ms: multi-scale.

Setups	Baseline	$F_{1}$	$F_{2}$	$F_{3}$	mIoU (ss)	mIoU (ms)	#params
Setup 1	*√*				53.9	55.1	107.0M
Setup 2	*√*	*√*			54.2 (↑0.3)	55.3 (↑0.2)	107.1M
Setup 3	*√*		*√*		54.3 (↑0.4)	55.3 (↑0.2)	107.3M
Setup 4	*√*			*√*	54.0 (↑0.1)	55.1 (-)	109.2M
Setup 5	*√*	*√*	*√*		54.3 (↑0.4)	55.3 (↑0.2)	107.4M
Setup 6	*√*	*√*		*√*	54.2 (↑0.3)	55.2 (↑0.1)	109.3M
Setup 7	*√*		*√*	*√*	54.3 (↑0.4)	55.3 (↑0.2)	109.5M
Setup 8	*√*	*√*	*√*	*√*	54.5 (↑0.6)	55.5 (↑0.4)	109.6M

**Table 2:** Performance comparison of various auxiliary CNNs using the ADE20K validation set. ss: single-scale. ms: multi-scale.

Auxiliary Structure	mIoU (ss)	mIoU (ms)	#params
-	53.9	55.1	-
1×1 conv.	53.6 (↓0.3)	54.7 (↓0.4)	1.4M
3×3 conv.	54.0 (↑0.1)	55.0 (↓0.1)	12.4M
one residual block	54.2 (↑0.3)	55.2 (↑0.1)	1.3M
two residual blocks	54.5 (↑0.6)	55.5 (↑0.4)	2.6M

**Table 3:** Performance comparison of different weighting parameters β using the ADE20K validation set. ss: single-scale. ms: multi-scale.

Weighting Parameter β	mIoU (ss)	mIoU (ms)
-	53.9	55.1
0.1	54.5 (↑0.6)	55.5 (↑0.4)
0.2	54.4 (↑0.5)	55.3 (↑0.2)
0.3	54.1 (↑0.2)	55.2 (↑0.1)
0.05	54.2 (↑0.3)	55.3 (↑0.2)

**Table 4:** Performance comparison of semantic segmentation on the ADE20K validation dataset with 150 categories. †: backbone pretrained on ImageNet-22K. ss: single-scale. ms: multi-scale.

Method	Backbone	Crop Size	mIoU (ss)	mIoU (ms)
PVTv1 [39]	PVTv1-L	512 × 512	44.8	-
PVTv2 [40]	PVTv2-B5	512 × 512	48.7	-
P2T [41]	P2T-L	512 × 512	49.4	-
Swin-UperNet [42,47]	Swin-L ^†^	640 × 640	-	53.5
FaPN-MaskFormer [10,48]	Swin-L ^†^	640 × 640	55.2	56.7
BEiT-UperNet [4,47]	BEiT-L ^†^	640 × 640	-	57.0
MaskFormer [10]	Swin-T	512 × 512	46.7	48.8
	Swin-S	512 × 512	49.8	51.0
	Swin-B ^†^	640 × 640	52.7	53.9
	Swin-L ^†^	640 × 640	54.1	55.6
Mask2Former [11]	Swin-T	512 × 512	47.7	49.6
	Swin-S	512 × 512	51.3	52.4
	Swin-B ^†^	640 × 640	53.9	55.1
	Swin-L ^†^	640 × 640	56.1	57.3
Mask2Former (Ours)	Swin-T	512 × 512	47.9	49.7
	Swin-S	512 × 512	51.3	52.5
	Swin-B ^†^	640 × 640	54.1	54.9
	Swin-L ^†^	640 × 640	56.0	57.1
Ours	Swin-T	512 × 512	48.8	50.3
	Swin-S	512 × 512	52.2	53.1
	Swin-B ^†^	640 × 640	54.5	55.5
	Swin-L ^†^	640 × 640	56.4	57.6

**Table 5:** Performance comparison of semantic segmentation on the Cityscapes validation dataset with 19 categories. †: backbone pretrained on ImageNet-22K. ss: single-scale. ms: multi-scale.

Method	Backbone	Crop Size	mIoU (ss)	mIoU (ms)
Segmenter [9]	ViT-L ^†^	768 × 768	-	81.3
SETR [7]	ViT-L ^†^	768 × 768	-	82.2
SegFormer [8]	MiT-B5	768 × 768	-	84.0
Mask2Former [11]	Swin-S	512 × 1024	82.6	83.6
	Swin-B ^†^	512 × 1024	83.3	84.5
	Swin-L ^†^	512 × 1024	83.3	84.3
Mask2Former (Ours)	Swin-S	512 × 1024	82.4	83.5
	Swin-B ^†^	512 × 1024	83.2	84.3
	Swin-L ^†^	512 × 1024	83.3	84.3
Ours	Swin-S	512 × 1024	82.9	83.8
	Swin-B ^†^	512 × 1024	83.8	84.8
	Swin-L ^†^	512 × 1024	83.6	84.5

**Table 6:** Performance comparison of panoptic segmentation on the ADE20K validation dataset. Single-scale (ss) inference is adopted by default. Multi-scale results are marked with *. †: backbone pretrained on ImageNet-22K.

Method	Backbone	Panoptic Model		
		PQ (ss)	AP_pan_	mIoU_pan_
BGRNet [34]	R50	31.8	-	-
Auto-Panoptic [35]	ShuffleNetV2 [49]	32.4	-	-
MaskFormer [10]	R50	34.7	-	-
Kirillov et al. [30]	R50	35.6 *	-	-
Panoptic-DeepLab [31]	SWideRNet [50]	37.9 *	-	50.0 *
Mask2Former [11]	Swin-L ^†^	48.1	34.2	54.5
Mask2Former (Ours)	Swin-L ^†^	48.3	34.0	54.4
Ours	Swin-L ^†^	48.8	35.1	54.7

**Table 7:** Performance comparison of panoptic segmentation on the Cityscapes validation dataset. Single-scale (ss) inference is adopted by default. Multi-scale results are marked with *. †: backbone pretrained on ImageNet-22K. ‡: backbone pretrained on ImageNet-1K and COCO.

Method	Backbone	Panoptic Model		
		PQ (ss)	AP_pan_	mIoU_pan_
TASCNet [32]	R50 ^‡^	59.2	-	-
Kirillov et al. [30]	R50	61.2 *	36.4 *	80.9 *
UPSNet [33]	R101 ^‡^	61.8 *	39.0 *	79.2 *
Panoptic-DeepLab [31]	SWideRNet [50]	66.4	40.1	82.2
Panoptic-FCN [36]	Swin-L ^†^	65.9	-	-
Mask2Former [11]	Swin-B ^†^	66.1	42.8	82.7
	Swin-L ^†^	66.6	43.6	82.9
Mask2Former (Ours)	Swin-B ^†^	65.7	42.8	82.1
	Swin-L ^†^	66.4	43.0	82.9
Ours	Swin-B ^†^	66.6	43.8	82.9
	Swin-L ^†^	66.7	44.6	83.2

## Data Availability

The data presented in this study are openly available in ADE20K at 10.1109/CVPR.2017.544, reference number [16] and Cityscapes at 10.48550/arXiv.1604.01685, reference number [17].
